# Molecular Weight Segregation and Thermal Conductivity of Polydisperse Wax–Graphene Nanocomposites

**DOI:** 10.3390/polym15092175

**Published:** 2023-05-03

**Authors:** Maarten Boomstra, Bernard Geurts, Alexey Lyulin

**Affiliations:** 1 Soft Matter and Biological Physics Group, Department of Applied Physics and Science Education, Eindhoven University of Technology, P.O. Box 513, 5600 MB Eindhoven, The Netherlands; 2 Center for Computational Energy Research, P.O. Box 6336, 5600 HH Eindhoven, The Netherlands; 3 Mathematics of Multiscale Modeling and Simulation, Department of Applied Mathematics, Faculty EEMCS, University of Twente, P.O. Box 217, 7500 AE Enschede, The Netherlands

**Keywords:** molecular dynamics modelling, phase change material, thermal conductivity, paraffin wax, graphene nanofiller, nanocomposite, polydispersity, segregation

## Abstract

Paraffin waxes are a promising material for heat storage with high energy density. Their low thermal conductivity, which limits the speed of charging and discharging in heat buffers, was previously shown to be improved by adding graphene nanofillers. In the present study, using molecular dynamics simulations, the segregation by molecular weight of polydisperse paraffin near graphene flakes is investigated. In liquid bidisperse paraffin composed of decane and triacontane, an aligned layer containing mainly triacontane was observed next to the graphene. Upon slow cooling, the wax crystallised into distinct layers parallel to the graphene sheet, with much stronger segregation by molecular weight than in the crystallised bidisperse wax without graphene. For polydisperse wax, the segregation effect was much less pronounced. The molten paraffin had a somewhat higher concentration of the longest chains in the first layers next to the graphene, but during crystallisation, the molecular weight segregation was only slightly increased. Measurements of crystallinity using an alternative version of the method developed by Yamamoto showed that the layers of wax were highly aligned parallel to the graphene, both in the solid state with all wax crystallised and in the liquid state with one layer of aligned wax above and below the graphene. Thermal conductivity was increased in planes parallel to the graphene flakes. The strong segregation of chain lengths in the bidisperse wax resulted in clear differences in thermal conductivity in the segregated regions. The less segregated polydisperse wax showed less variation in thermal conductivity.

## 1. Introduction

An important prerequisite for the ongoing energy transition is the storage of energy [1]. This holds for electricity, which is supplied less constantly from renewable sources such as the sun and wind than from fossil fuels, but equally so for thermal energy. The fast storage and retrieval of large amounts of heat with little loss is essential for environmentally friendly thermal energy management and is one of the main challenges to solve regarding the current energy crisis [2,3]. A promising group of substances for thermal energy storage is phase change materials (PCM), which make use of latent heat. PCMs absorb or release heat during a thermodynamic phase transition and therefore have a high energy density [4,5]. PCMs are commonly classified as organic, inorganic or eutectic (which are combinations of two or more PCMs) [2].

One specific example of an organic PCM material is paraffin wax [2]. Because of their convenient melting range at roughly body temperature, these are very suitable for use in domestic heating [3]. Paraffin wax stores heat effectively due to its large latent heat of roughly 130 kJ/kg [6]. An important downside is that paraffin has low thermal conductivity (TC) in the range of 0.1–0.3 W/(m K). The main reason for paraffin’s low TC lies in the fact that heat is predominantly transported through the backbone [7,8]. Therefore, crystallised paraffin, in which the alignment of backbones is present, has a higher TC than its molten counterpart [9]. The low TC means that heat cannot be transported through the paraffin quickly, and therefore fast storage into and retrieval from heat buffers based on paraffin alone is not possible [10].

Many previous studies, both experimental [6,11] and modelling [12,13], have shown that the TC of paraffin wax can be improved with graphene nanofillers. Graphene, a sheet of graphite composed of one or a few layers, has attracted much attention because of its unique electronic properties [14] and extremely high thermal conductivity of 2000–4000 W/(m K) [15]. Adding graphene to paraffin, making a nanocomposite, enhances TC as compared to pristine paraffin. For example, Fan et al. [11] measured an increase in TC of 164% when 5 wt% of graphene nanoplatelets (GNP) was added. Babaei et al. [16] showed, with molecular dynamics (MD) computer simulations, that the presence of graphene fillers increases the TC of pristine paraffin, by promoting the ordering of the paraffin chains. At a mass fraction of 1.55%, they measured an increase in TC of 64% for the solid wax in the composite.

Compared to the TC of graphene (2000–4000 W/(m K) [15]), the obtained TCs of graphene–paraffin composites, 0.49 W/(m K) by Babaei et al. and 0.7 W/(m K) by Fan et al., are still very low, and only two to three times higher than that of the pure wax. For practical use in heat storage applications, a further increase in the TC is required [3]. This paper contributes to understanding the basic partial ordering in the paraffin–graphene system and how this affects the value of the TC.

Many studies have been devoted to establishing a link between the crystallinity and thermal conductivity of polymers. For example, Borhani et al. [17] have studied the effect of crystallinity and irradiation on the thermal properties of low-density polyethylene under various cooling rates. They showed that increasing the cooling rate leads to a drastic decrease in crystallinity, which is accompanied by a corresponding TC decrease. Using large-scale MD modelling, Zhang et al. [7] found that polymer nanofibers consisting of molecules with rigid backbones have outstanding TC because of the long phonon mean free paths due to reduced disorder scattering. Similar conclusions were drawn by Chen et al. [8], who showed that crystallinity and orientation strongly influence TC. The sizes of the regions in which molecules are aligned therefore play an important role in the overall TC of the polymer material. A central question that is decisive for the prediction of the TC value of the paraffin–graphene nanocomposite is how the presence of graphene fillers influences the paraffin crystallisation. We can also address the feasibility question of whether atomistic MD simulations are useful to study these phenomena in view of the usually slow polymer crystallisation and extremely high MD cooling rates. In our previous publication [9], we showed the strong relation between the crystallinity and the TC of pure paraffin waxes. For polydisperse waxes with various molecular weight distributions, no strong differences in crystallisation were measured as compared to monodisperse eicosane samples [9]. This finding is in agreement with the results of Luo et al. [18], who performed MD simulations of length-dependant segregation in n-alkanes. They concluded that hardly any segregation takes place in polydisperse mixtures, and the crystallisation is therefore very similar to that of monodisperse parafin wax. However, Luo et al. also saw that during crystallisation, strong segregation takes place in bidisperse mixtures.

Zeng and Khodadadi [19] have carried out MD simulations of mixtures of n-eicosane (C20) and n-triacontane (C30), comparing paraffin in bulk to a set-up where a surface potential was introduced at the bottom of the simulation box. Such a potential can be considered as an implicit, non-structured surface of a nanofiller. In [19], it was shown that when introduced in this way, the surface potential results in a layered polymer structure in the solid phase, which affects the TC of the wax. No effect of variations in the C20/C30 ratio on the TC was measured. Moreover, no distinct segregation of chains with different molecular weights was observed.

In their theoretical studies, Wu et al. [20] and Hariharan et al. [21] looked into surface effects on polymer melts at neutral interfaces. Wu et al. found, using an extended self-consistent field theory, that the concentration of chain ends is higher at the surface. Hariharan et al. [21] researched chain length segregation at the surface of a polymer with a bimodal molecular weight distribution, concluding that the shorter molecules have a higher presence at the surface, as well as a preferential orientation perpendicular to the surface.

The question of the molecular weight segregation of polydisperse polymer samples in the presence of nanofillers is still an open issue. It is well known that longer polymer chains have higher crystallisation temperatures [22,23]. Upon cooling, nanofiller surfaces such as graphene flakes could induce the crystallisation of long chains in their proximity at an even higher temperature. In the case of polydisperse paraffin waxes, this potential molecular weight segregation can strongly influence the TC of the final graphene–paraffin composites.

Computational studies on the influence of paraffin–nanofiller interfaces were done by, e.g., Tong et al. [24]. They performed MD simulations of n-eicosane (using the united atom NERD force field for the wax) confined between sheets of graphene, showing that solid–liquid phase changes take place at much higher temperatures than in bulk, and that there is a distinct layering in the solid wax induced by graphene. Their study concerned only monodisperse wax, however, and TC was not measured. Experimental studies on the effect of confinement on the crystallisation and TC of paraffin were performed by, e.g., Deng et al. [25] and Fu et al. [26]. Using differential scanning calorimetry (DSC) and X-ray diffraction (XRD), Fu et al. [26] showed that mixtures of n-octadecane and n-eicosane crystallise differently in micro-encapsulation than in bulk. They found the surface freezing, as described earlier by Ocko et al. [27], to be enhanced, while also the lattice constants of the crystalline layer were changed as compared to the bulk. Deng et al. [25] measured the effect of the nano-confinement of heneicosane, also using DSC and XRD. They concluded that the melting and solidification temperatures are depressed and that the effects increase with decreasing pore size.

Paraffin waxes used in practice usually are polydisperse. Knowing more about the interaction of polydisperse paraffin with graphene will provide insights into the behaviour of the composite material during melting–solidification cycles and enable further optimisation for use in heat storage. Using MD modelling, the interactions between paraffin wax and graphene fillers can be studied at the molecular scale, thereby giving information unobtainable by experiments. This is what we try to achieve in the present study.

In the current paper, the influence of the graphene nanofiller on the segregation and resulting TC of the paraffin wax has been investigated. Our main goal is to have computational insights into the possible effects of the atomistically resolved nanofiller surfaces on the induced crystallisation, chain-length segregation and thermal conductivity for monodisperse and polydisperse paraffin-based nanocomposites. Segregation is quantified as the number of atoms belonging to molecules of each chain length per layer and the TC is measured by equilibrium MD (EMD), making use of the Green–Kubo formalism [28,29], as well as reverse non-equilibrium MD (RNEMD) [30].

The article is structured as follows. In Section 2, the models and the details of the MD simulations and computational measurement techniques are discussed. In Section 3, the results for the molecular weight segregation and TC of paraffin–graphene nanocomposites are given. Conclusions are formulated in Section 4.

## 2. Models and Methods

In this section, we first consider the applied models for the paraffin–graphene composites and discuss measurement techniques afterwards.

All simulations in the present study were performed with the fully atomistic Generalised Amber Force Field [31] (GAFF). This was previously shown to give the best results when measuring the TC of paraffin by Nazarychev et al. [32]. Paraffin molecules as well as graphene sheets were generated using AmberTools20 [33,34] and converted into GROMACS [35,36] input files using the AnteChamber PYthon Parser interface [37] (ACPYPE). Composites of graphene with three types of paraffin wax were produced: monodisperse, bidisperse and polydisperse. All samples contained 900 paraffin chains, comprising purely eicosane C${}_{20}$H${}_{42}$ for the monodisperse wax, 450 chains of decane C${}_{10}$H${}_{22}$ and 450 chains of triacontane C${}_{30}$H${}_{62}$ for the bidisperse wax and a distribution of chain lengths as specified in our previous study [9] for the polydisperse wax. The graphene sheet contained 3680 carbon atoms and was simulated as having an infinite area by crossing the periodic boundary conditions in the x- and y-directions. To prevent the folding of the infinite graphene sheet, the x- and y-dimensions of the simulation box were always kept constant.

The paraffin molecules of different lengths needed to be produced separately, using AmberTools20 as described above. By combining the desired number of chains of each length, an initial configuration of the wax was produced that was very structured and had strong molecular weight segregation. To ensure a total loss of these, the paraffin was heated up to 600 K in the NVT ensemble, at which it was kept for 2 ns and then rapidly cooled down to 300 K. After adding the graphene sheet and the wax together, the composite was again heated to 600 K, held at this temperature for 2 ns and then rapidly cooled down to 450 K. This was done using the Berendsen thermostat and velocity rescale barostat at a pressure of 1 atm. Only the length of the box in the z-direction was allowed to change.

Timesteps were set to 2 fs and C-H bonds were constrained using the LINCS [38] algorithm. For pure paraffin, previous studies [9,32,39] showed that a cooling rate of 0.1 K/ns allows for crystallisation and leads to a density in a solid state that agrees reasonably with experimental values. In the present study, molecular weight segregation in the bidisperse and polydisperse samples took place during crystallisation. Cooling at 0.1 K/ns allowed this to occur and, as discussed in the Supplemental Information, lower cooling rates did not lead to significantly different crystallisation. The cooling rate of 0.1 K/ns was therefore implemented, keeping the total simulation time manageable. However, this resulted in large times needed to cool the samples down. For example, the longest simulations, of composites with polydisperse wax cooled from 450 K to 200 K, took one and a quarter billion timesteps, corresponding to roughly 2 million seconds of physical time for their simulation on a parallel infrastructure using 64 cores.

GROMACS [35,36] was used for the cooling because of its speed, but it lacks the possibility of TC measurement directly during simulation. After cooling, the simulation files were therefore transferred to LAMMPS [40] to make use of the latter’s built-in TC measurement functions. Transfer was executed using InterMol [41]. Prior to measurements, all samples were equilibrated in the NVT ensemble using the Nosé–Hoover thermostat for 1 ns, followed by 1 ns of equilibration in the NVE ensemble. Timesteps were set to 0.5 fs and no constraints of C-H bonds were applied.

As will be seen in Section 3, the presence of a sheet of graphene induces the crystallisation of paraffin in layers parallel to the graphene. To look further into the properties of these layers, measurements localised in sub-domains of one or a few paraffin molecules in thickness were done.

Molecular weight segregation was quantified using relative densities. In each layer, the number density of atoms that were part of a chain of a specific length was determined. Dividing this by the average density (over the full simulation box) of the molecular weight under consideration, the relative density was obtained. This gives an indication of how segregated the chains of respective molecular weights are.

**Figure 1:**
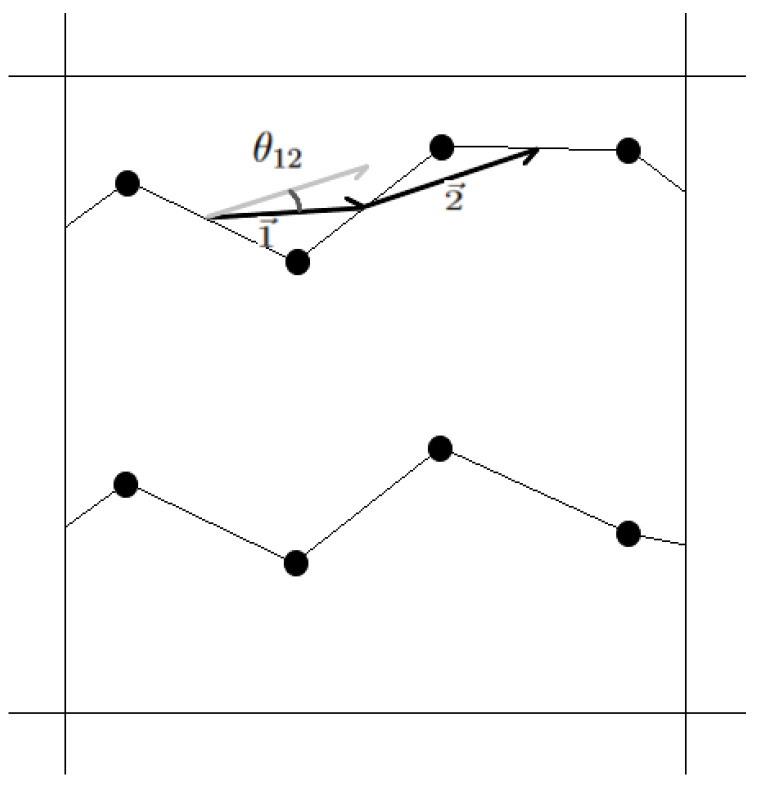
Schematic drawing of chord vectors used in the measurement technique by Yamamoto [42]. The top view of a sub-layer is shown in which parts of two molecules are present. Only carbon–carbon bonds are considered in this technique. Chord vectors named $\vec{1}$ and $\vec{2}$ connect the midpoints of neighbouring carbon bonds. $\theta_{12}$ is the angle between these two chord vectors.

Crystallinity was measured with the method developed by Yamamoto [42], altered slightly to obtain the per layer crystallinity. Instead of dividing the simulation box into sub-domains of roughly ${\left(2\sigma \right)}^{3}$ [42] with σ the Lennard–Jones distance parameter (in this case, of carbon–carbon interactions), each layer is divided into sub-layers of an area roughly equal to ${\left(2\sigma \right)}^{2}$. A schematic depiction is shown in Figure 1. Per sub-layer, the alignment of all chord vectors, which connect the mid-points of adjacent carbon–carbon bonds, is quantified by the orientational order parameter $P_{2}$, defined using Legendre polynomials of the second order,

(1) $$P_{2}=\langle 3\;\cos^{2}\left(\theta_{ij}\right)-1\rangle /2,$$

with $\theta_{ij}$ being the angle between chord vectors $\vec{i}$ and $\vec{j}$ within the same sub-domain. The crystallinity of a layer is then defined as the fraction of sub-layers in which the alignment of the chord vectors $P_{2}$ is above the threshold value 0.7 as suggested by Yamamoto [42]. In addition to this, the alignment of all chord vectors contained in a layer, with the z-axis, is measured by

(2) $$P_{z}=\langle 3\;\cos^{2}\left(\phi_{i}\right)-1\rangle /2,$$

with $\phi_{i}$ as the angle between chord vector $\vec{i}$ and the z-axis. The lowest possible value, −0.5, indicates full perpendicularity to the z-axis, and therefore full alignment with the xy-plane.

To enable TC measurement per region, the equilibrium molecular dynamics (EMD) measurement technique using the Green–Kubo [28,29] formalism is the most obvious choice. Since these measurements are performed in equilibrium, a specific zone can be chosen in which to measure TC, unlike non-equilibrium techniques in which a temperature gradient is present. The heat flux is defined as

(3) $$\begin{array}{cc} \vec{J} & =\frac{1}{V}\left[\sum_{i}e_{i}{\vec{v}}_{i}-\sum_{i}S_{i}{\vec{v}}_{i}\right]\\ \; & =\frac{1}{V}\left[\sum_{i}e_{i}{\vec{v}}_{i}-\sum_{i<j}\left({\vec{F}}_{ij}\cdot {\vec{v}}_{j}\right){\vec{r}}_{ij}\right], \end{array}$$

with *e*${}_{i}$ as the per atom energy, ${\vec{v}}_{i}$ is the velocity vector of particle i, ***S***${}_{i}$ the per atom stress tensor, ${\vec{F}}_{ij}$ is the force of particle i on particle j and ${\vec{r}}_{ij}$ is the vector connecting these particles. TC is then calculated by integrating the autocorrelation function of the heat flux over time,

(4) $$\kappa =\frac{V}{3k_{B}T^{2}}\int_{0}^{\infty }\langle \vec{J}\left(0\right)\cdot \vec{J}\left(t\right)\rangle dt,$$

with *V* [m${}^{3}$] as the volume of the region in which TC is measured and *T* [K] the temperature. For ease of reading, only the definition of heat flux valid for two-body interactions is shown in Equation (3). For this study, the March 2020 version of LAMMPS was used, in which heat flux for many-body interactions is calculated using the corrections by Boone et al. [43] with the *compute centroid/stress/atom* function.

Additionally, the reverse non-equilibrium MD (RNEMD) technique developed by Müller-Plathe [30] was used for TC measurements. In this method, the simulation box is divided into an even number *N* of slabs. By interchanging the velocities of the coldest atom in the central slab—more specifically, slab number N/2 + 1 with N the total number of slabs—and the hottest atom in the first slab (at the edge of the simulation box), a temperature difference is induced. Once a steady state is reached, the heat flowing from the hot central slab to the cold outer slab is equal to the swapped kinetic energy,

(5) $$J=\frac{\sum_{\mathrm{transfers}}\frac{m}{2}\left({v_{h}}^{2}-{v_{c}}^{2}\right)}{2tL_{x}L_{y}},$$

with *J* [W/m${}^{2}$] the heat flux, *v*${}_{h}$ and *v*${}_{c}$ [m/s] the velocities of the hottest and coldest particles with mass *m* [kg] at each transfer, *t* [s] the total time of measurement and *L*${}_{x}\;\times$ *L*${}_{y}$ the area [m${}^{2}$] through which the heat flux flows, in this case parallel to the z-axis. The factor 2 comes from the heat flowing in two directions, to slab 1 and its periodic copy at N + 1. With the heat flux known and after measuring the temperature difference, the TC can be calculated using Fourier’s law,

(6) J=−κ∇T

with *J* the heat flux [W/m${}^{2}$], κ TC [W/(m K)] and ∇T [K/m] the temperature gradient. To measure TC per region, swapping of the velocities was only done in slabs in the region under consideration. Furthermore, only the positions of atoms in the region of interest were updated during TC measurements, while all other atoms were immobilised. In this way, the participation of atoms outside the respective region to heat transport causing incorrectly high TC values was prevented.

## 3. Results and Discussion

### 3.1. Effect of Graphene Sheets on Molecular Weight Segregation in Paraffin

**Figure 2:**
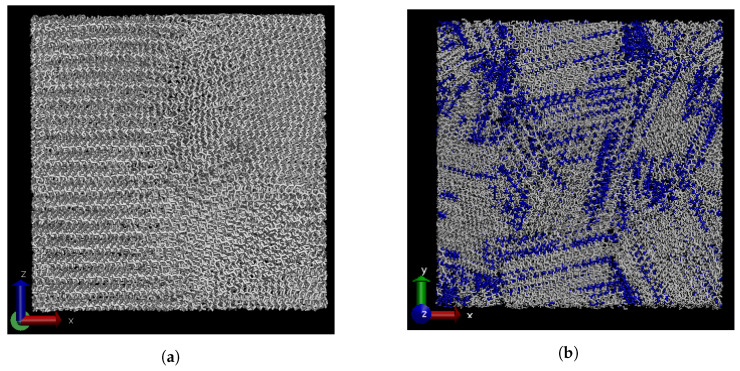
Monodisperse eicosane (**a**) and bidisperse wax containing the same number of C10 and C30 molecules shown in blue and white, respectively (**b**), both at 250 K. The simulation boxes contain 900 molecules and measure roughly 8×8×8 nm${}^{3}$ in size. Zones with different orientations of the chains are visible in both cases. In the bidisperse wax, partial segregation of the chain lengths is observed. Decane chains are depicted in blue.

In our previous publication [9], the TC of paraffin wax well above (450 K) and below (250 K) the melting temperature was studied. During crystallisation, regions with different orientations of the molecules were formed side by side. An example of semi-crystalline monodisperse eicosane is shown in Figure 2a.

Luo et al. [18] looked into the molecular weight segregation of bidisperse and polydisperse paraffin by quenching. While, for the polydisperse samples, they observed only weak segregation, the bidisperse wax did show clear segregation of chain lengths. In the current study, we used a much lower cooling rate of 0.1 K/ns. To check if the same results are obtained using this cooling technique, a mixture of 450 decane and 450 triacontane molecules was cooled down. Figure 2b shows that multiple regions of segregated molecular weights were formed, similar to those found by Luo et al. [18].

**Figure 3:**
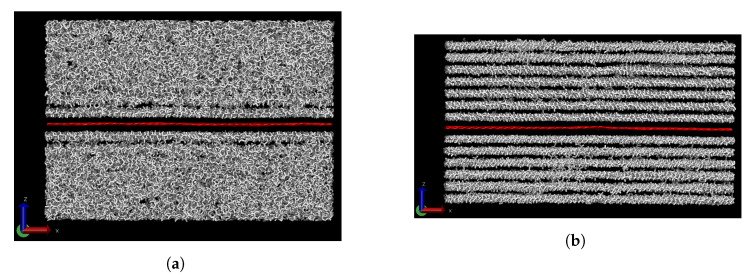
Monodisperse eicosane with a sheet of graphene (red) at 450 K (**a**) and 350 K (**b**). A first aligned layer of eicosane is seen next to the graphene at 450 K. At 350 K, all the wax is crystallised and has formed distinct layers.

The effect of the presence of carbon nanofillers such as graphene on the thermal conductivity of monodisperse paraffin wax was studied, e.g., by Babaei et al. [16,44]. They showed that the paraffin closest to an infinite graphene sheet forms an aligned layer even when the rest of the paraffin is still in a liquid state. Upon cooling, further alignment results in the formation of distinct layers. As a consequence of this, the TC of the paraffin along these planes is enhanced. In Figure 3, similar results are observed, i.e., a first layer formed in the melt and multiple distinct layers in the solid. The presence of graphene will also influence the chain length segregation of bidisperse and possibly of polydisperse wax. This will be considered next.

**Figure 4:**
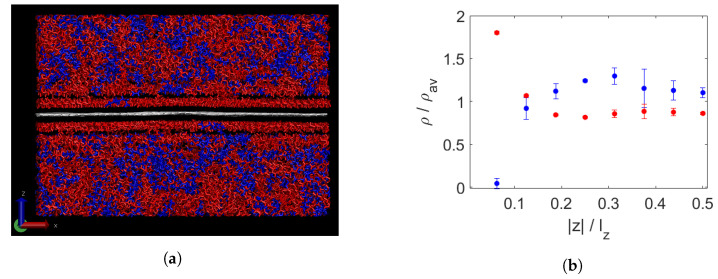
Bidisperse wax and a sheet of graphene (white) at 450 K (**a**). Long chains are shown in red, short ones in blue. Segregation per layer, measured by normalised density per chain length (**b**). On the x-axis, the distance from the graphene sheet, divided by the box height l${}_{z}$, is adopted. The liquid part of the wax is divided into slabs of equal thickness to that of the first layer above and below the wax. These first layers consist of almost pure triacontane (red) and have a higher total denisity than the bulk fluid.

Figure 4a shows a composite of graphene with an even mixture of decane and triacontane. An aligned layer above and below the graphene can be distinguished even above the melting temperature, which seems to be composed mainly of triacontane. In Figure 4b, the segregation is quantified by means of relative densities, as described in Section 2. Indeed, we see that there is almost no decane present in the layer closest to the graphene. Furthermore, it is observed that the density of triacontane in this layer is enhanced due to the induced alignment of the molecules. Slightly further away, measured as a fraction of the computation box, there is still a somewhat lowered relative density of decane, while that of triacontane is slightly increased. In the rest of the molten wax, the remaining molecules are distributed without large density fluctuations and with some asymmetry, shown by the error bars.

**Figure 5:**
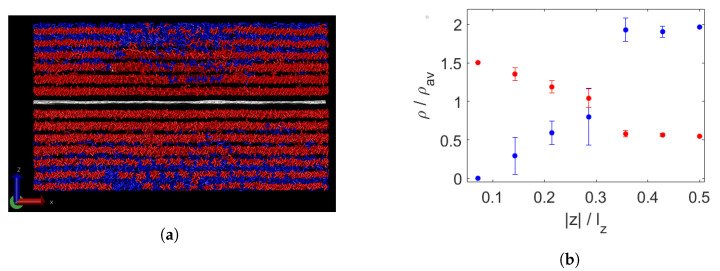
Bidisperse wax and a sheet of graphene (white) at 350 K (**a**). Long chains are shown in red, short ones in blue. The triacontane molecules have crystallised into layers. Segregation per layer, measured by normalised density per chain length (**b**). On the x-axis is the distance from the graphene sheet, divided by the box height l${}_{z}$. The relative density of triacontane (red) decreases steadily in the closest four layers above and below the graphene, while that of decane (blue) increases. At a larger distance from the graphene, in the furthest three layers above and below, the relative densities are nearly constant. The large error bar for the fourth layer indicates asymmetry between the layers at the top and bottom sides of the graphene.

In Figure 5, the results of the bidisperse composite at 350 K are shown. At this temperature, all triacontane has crystallised into layers, while the decane is still liquid. Segregation seems strongest in the layers closest to the graphene. This is partially confirmed in Figure 5b, which shows a gradual decrease in segregation with increasing distance to the graphene in the closest four layers on each side. In the wax furthest away, the relative densities are nearly constant. Here, many decane chains are present while the density of triacontane is rather low.

**Figure 6:**
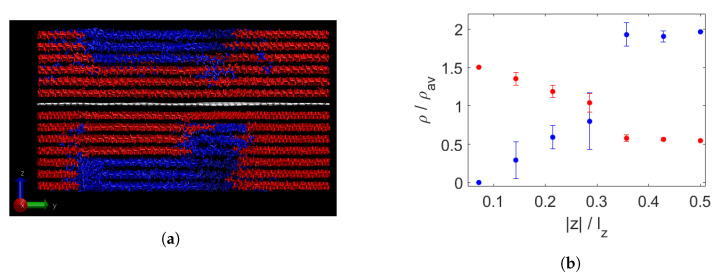
Bidisperse wax and a sheet of graphene (white) at 250 K (**a**). Long chains are shown in red, short ones in blue. In addition to the triacontane chains that had crystallised already at 350 K, now also the decane molecules have crystallised into layers. Segregation per layer, measured by normalised density per chain length (**b**). On the x-axis is the distance from the graphene sheet, divided by the box height l${}_{z}$. The segregation has not changed compared to the semi-solid situation at 350 K shown in Figure 5b.

Upon further cooling to 250 K, the decane has also crystallised into clear layers, as shown in Figure 6a. Segregation seems to be into a single separate region for each molecular weight. In Figure 6b, no difference is observed with Figure 5b, from which it can be concluded that all segregation took place during the crystallisation of the triacontane.

**Figure 7:**
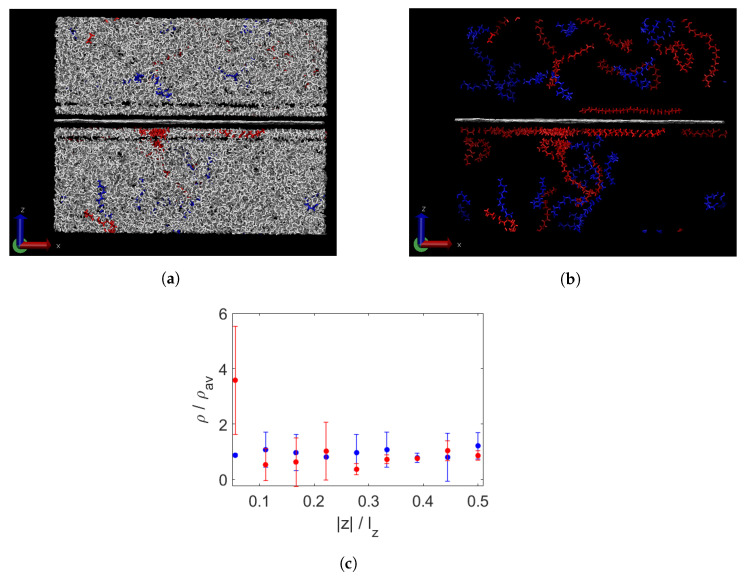
Polydisperse wax and a sheet of graphene (in the centre) at 450 K. All wax shown with the longest chains in red and shortest chains in blue (**a**) and only these chains and the graphene (white) (**b**). Segregation per layer, measured by normalised density per group of chain lengths (**c**). On the x-axis is the distance from the graphene sheet, divided by the box height l${}_{z}$. There is some agglomeration of long chains (red) near the graphene. The distribution near the graphene sheet displays considerable asymmetry, as indicated by the large error bar.

The composite containing polydisperse wax at 450 K is shown in Figure 7a. Some agglomeration of the longest chains is visible, seen more clearly in Figure 7b, where only the longest (red) and shortest (blue) chains are depicted. This is confirmed by the quantification in Figure 7c. These results are in agreement with the findings of Luo et al. [18], who found weaker segregation in polydisperse paraffin as compared to bidisperse paraffin.

**Figure 8:**
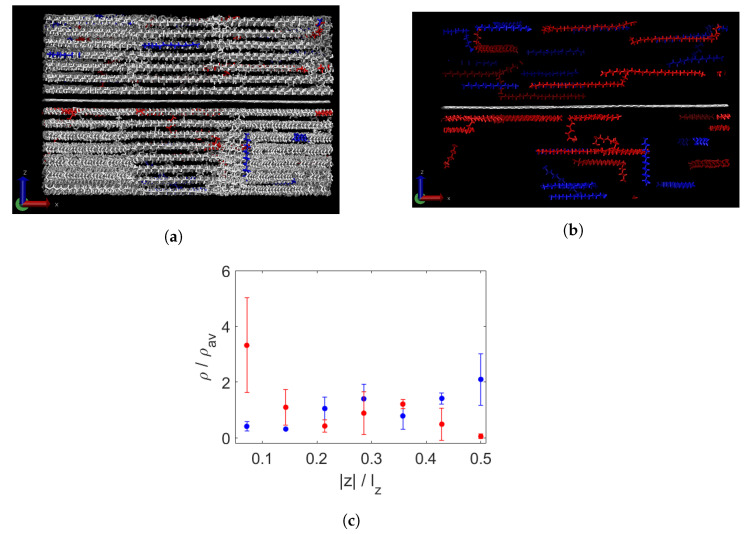
Polydisperse wax and a sheet of graphene (centre) at 200 K. All wax shown with the longest chains in red and shortest chains in blue (**a**) and only these chains and the graphene (white) (**b**). The wax has formed horizontally aligned layers. A few (partial) chains are not parallel to the graphene. Segregation per layer, measured by normalised density per group of chain lengths (**c**). On the x-axis is the distance from the graphene sheet, divided by the box height l${}_{z}$. There is no clear additional segregation compared to the situation at 450 K, except for the layers furthest from the graphene, in which the density of the longest chains (red) is clearly lowered while that of the short chains (blue) is increased.

In Figure 8a, the same polydisperse sample is shown, now at 200 K. A clear layering is apparent, although some of the highlighted chains in Figure 8b are (partially) in a non-horizontal alignment. However, not much additional segregation is measured after cooling except in the layer furthest from the graphene.

In conclusion, a strong segregation was observed in the bidisperse composite. The presence of the graphene sheet induced the formation of much larger regions with a single chain length than was seen in the bidisperse wax without graphene. In the polydisperse composite, some segregation was present in the molten state. However, upon cooling, this did not increase as substantially as for the bidisperse wax. In comparison to the studies by Wu et al. [20] and Hariharan et al. [21], these results are the complete opposite. In these studies, at a neutral surface, the concentration of chain ends and short molecules, respectively, was increased, which was explained by entropic effects. The segregation observed in the present study seems to be caused by the lower crystallisation temperature of the long paraffin molecules than that of short molecules, as concluded for pure wax by Luo et al. [18], and this effect was amplified by the presence of the graphene.

### 3.2. Crystallinity

**Figure 9:**
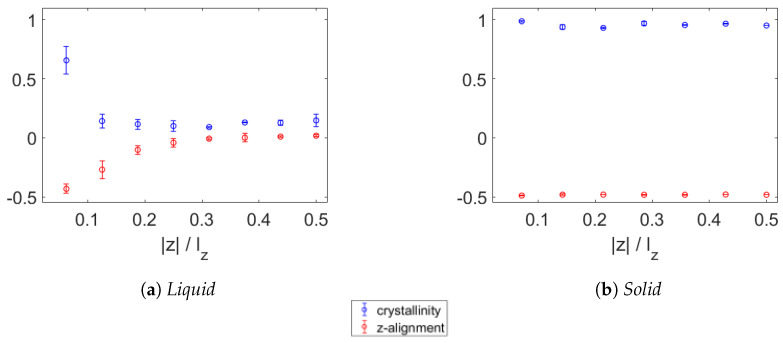
Crystallinity (blue) and alignment with z-axis (red) of monodisperse wax near a sheet of graphene in molten state at 450 K (**a**) and solid state at 350 K (**b**). At 450 K, only the layers directly above and below the graphene have increased crystallinity. The perpendicularity to the z-axis is low in the four layers closest to the graphene, two above and two below. In the solid state at 350 K, all layers have high crystallinity as well as strong alignment in the xy-plane.

Apart from the segregation observed in bi-/polydisperse paraffin during cooling near a graphene sheet, the formation of crystalline structures is a striking aspect induced by the presence of the graphene sheet. This will be investigated next. Using a slightly altered version of the technique developed by Yamamoto [42], the crystallinity of the various samples was measured per layer. In the case of molten wax, the box was divided into slabs with the same thickness as the aligned layer next to the graphene. In Figure 9, the crystallinity of the monodisperse eicosane wax is shown together with the alignment P${}_{z}$ of the molecules with the z-axis. In the molten state at 450 K shown in Figure 9a, the first layers above and below the graphene have significant crystallinity. The same goes for the z-alignment, which is still rather small (close to the minimum of −0.5) in the second layers from the graphene. At 350 K, as plotted in Figure 9b, all layers of wax have high crystallinity as well as large perpendicularity to the z-axis. Previous studies have shown that, because heat transport takes place mainly in the backbone [45], the TC increases with crystallinity and alignment [9,22]. It is therefore expected that the wax will have an enhanced TC in the xy-plane and a lowered TC in the z-direction, as will be discussed later.

**Figure 10:**
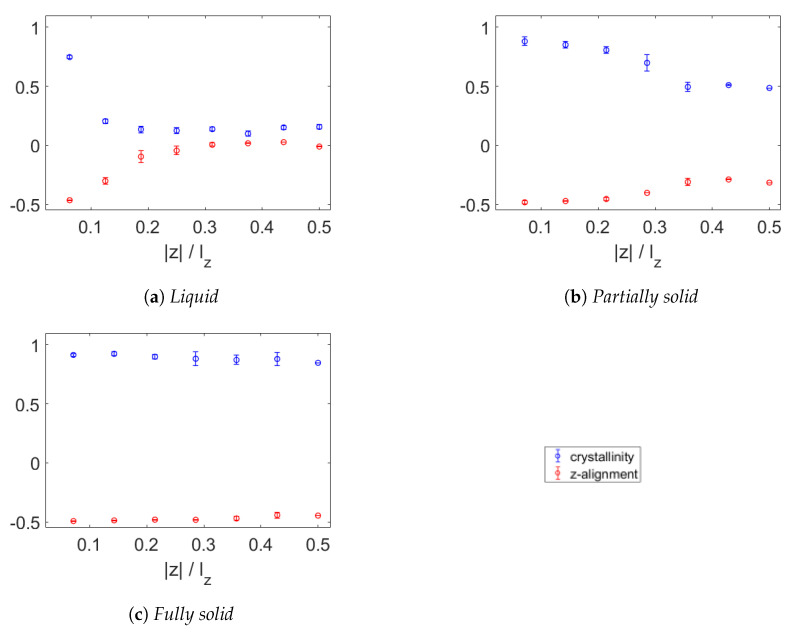
Crystallinity (blue) and alignment with z-axis (red) of bidisperse wax near a sheet of graphene in liquid state at 450 K (**a**), semi-solid state at 350 K (**b**) and fully solid state at 250 K (**c**). In the liquid state, only the first layers have increased crystallinity, The first two are noticeably unaligned with the z-axis. At 350 K, more layers have high crystallinity and alignment with the xy-plane. There is a gradual decrease in these properties with increasing distance from the graphene. The furthest 3 layers have nearly constant values. At 250 K, all the wax has crystallised into layers parallel to the xy-plane.

In Figure 10, the temperature dependence of the crystallinity is presented. The bidisperse wax at 450 K, seen in Figure 10a, shows similar crystallinity and z-alignment to those of the monodisperse wax at the same temperature, displaying high crystallinity only in the direct vicinity of the graphene sheet. In Figure 10b, showing the results at 350 K, high crystallinity is seen for the layers closest to the graphene, while the furthest layers have crystallinity of only roughly 0.5. This confirms what was observed earlier, as only the triacontane has crystallised at 350 K and the decane is still liquid. Upon cooling to 250 K, as shown in Figure 10c, all wax has crystallised and aligned almost fully in the xy-plane.

**Figure 11:**
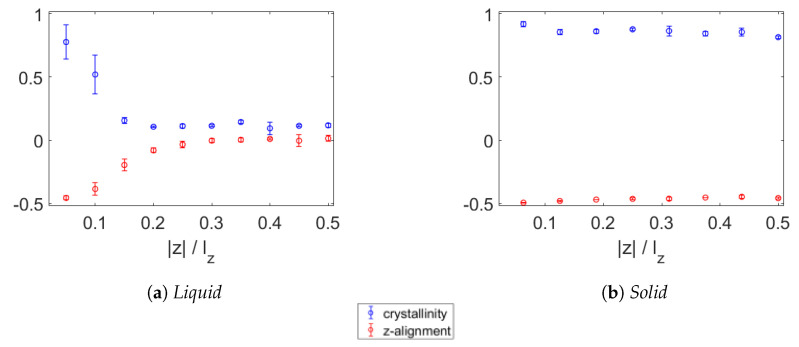
Crystallinity (blue) and alignment with z-axis (red) of polydisperse wax near a sheet of graphene in molten state at 450 K (**a**) and solid state at 200 K (**b**). In the molten wax, the two layers above and two layers below the graphene have increased crystallinity compared to the bulk liquid. The alignment with the xy-plane is increased in the first three layers above and below the graphene. At 200 K, the paraffin is highly crystalline and nearly perpendicular to the z-axis.

In Figure 11, the crystallinity and alignment with the z-axis are shown for the polydisperse wax. At 450 K, as seen in Figure 11a, the first two layers above and below the graphene have a high crystallinity while the first three layers are (partially) aligned parallel to the graphene sheet. At 250 K, as shown in Figure 11b, all the wax is crystalline and perpendicular to the z-axis.

From previous studies [9,45], it is known that the transport of heat in paraffin takes place mainly along the backbones of the paraffin molecules. Better alignment in crystalline wax therefore results in a higher TC as compared to that of paraffin in the liquid state [22]. The presence of layers of wax parallel to the graphene sheet, as observed in the present study, is expected to have consequences for the TC of the aligned wax.

### 3.3. Thermal Conductivity

Since, in the crystalline state, almost all molecules are perpendicular to the z-axis, it is expected that the z-component of TC will be reduced while heat transport along the xy-plane will be enhanced. Furthermore, it was shown previously [9,22,45,46,47] that paraffin of higher molecular weight and with high alignment has a higher TC. The strong segregation in the crystallised bidisperse composite is therefore likely to cause a higher TC in the layers with a high triacontane concentration.

The most obvious method to measure TC appears to be the EMD technique using the Green–Kubo [28,29] formalism. Because these measurements take place in equilibrium, it should be easy to determine the TC of a region. This method was applied to the simulation findings and gave the qualitative insight that the z-component of the TC was much lower than the x- and y-components in the solid wax and in the layers closest to the graphene sheet. This is in agreement with expectation because of the strong alignment within the xy-plane.

Further TC measurements were done with the RNEMD technique developed by Müller-Plathe [30], as described in Section 2. The results are shown in Table 1, which contains the averages and standard deviations of the x- and y-components of TC per region. The z-components could not be obtained with this technique due to highly non-linear temperature profiles in the z-direction, caused by the layering of the wax. In the eicosane in the liquid state, an increased TC is observed in the layers closest to the graphene. This agrees with expectation because of the increased crystallinity and alignment of these layers. The isotropy of the remaining paraffin is seen in the low standard deviation of 0.01 W/(m K). In the solid state, the TC is increased by further crystallisation. However, a decrease is seen in the layers further from the graphene in the solid state. This is caused by the constant area of the xy-plane during cooling, leading to the formation of a small void in the crystalline wax further from the graphene, as further discussed in the SI.

In the bidisperse composite, the same effects are observed. In the liquid state, the aligned layers close to the graphene have an increased TC compared to the fully liquid paraffin, which has a very low standard deviation in TC due to isotropy. In the semi-solid state, in which the triacontane has crystallised close to the graphene while the decane, which is concentrated further away, is still in a liquid state, there is almost a factor 2 difference in the TC of these regions. This agrees with expectation since the molecular weight and alignment are known to increase the TC [9,22,45,46]. After further cooling, the TC of the first four layers closest to the graphene sheet is roughly the same as in the semi-solid state, which was expected since these layers consist of mainly triacontane and the wax in these layers was almost fully crystallised already in the semi-solid sample, as shown in Figure 5b and Figure 10b. Similar to the solid monodisperse sample, the fully solidified bidisperse composite has a lowered TC in the furthest layers due to the formation of a void during crystallisation.

The polydisperse composite shows smaller differences in TC between the liquid and solid states. The molten paraffin at a large distance from the graphene shows isotropy, indicated by the very small standard deviation. The two layers closest to the graphene, the first of which has a significantly higher concentration of long molecules, as shown in Figure 8, and all of which have increased crystallinity and alignment in the xy-plane, have only a slightly increased TC compared to the fully liquid paraffin further away from the graphene, due to these layers only partially consisting of longer molecules. In the solid state, the TC is elevated compared to the liquid state due to the full crystallinity of the wax.

In summary, in the liquid samples, alignment of the paraffin closest to the graphene sheet results in a higher TC compared to the remaining paraffin. The strong molecular weight segregation of the bidisperse wax during crystallisation results in a factor 2 difference in TC comparing the regions containing mostly long and mostly short molecules, respectively. The differences in the polydisperse paraffin were much less pronounced due to the less pronounced differences in composition.

## 4. Conclusions

Paraffin waxes are a promising phase change material for heat storage, but significant improvement in their thermal conductivity is needed to enable fast charging and discharging. In the present study, we investigate the influence of a graphene nanofiller on the chain length segregation of polydisperse paraffin and the corresponding changes in TC. Making use of fully atomistic molecular dynamics simulations, the molecular weight segregation of paraffin as a consequence of the presence of graphene in the paraffin matrix, and the effect of this segregation on the thermal conductivity of the paraffin, were studied. With the paraffin in a molten state, a distinct layer above and below the graphene in which the paraffin molecules were highly aligned parallel to the graphene sheet was observed.

Measurements of relative densities showed that this layer consisted mainly of the longer chains in the bidisperse paraffin. For the polydisperse paraffin, this effect was also observed, but found to be less pronounced, in agreement with the findings by Luo et al. [18] for paraffin without graphene. After slow cooling, the paraffin wax formed into distinct layers. Relative density measurements showed the strong segregation of chain lengths in the bidisperse wax, formed during the crystallisation of the long chains, while, in the polydisperse wax, little change in relative densities was observed in comparison to the molten paraffin.

A slightly modified version of the crystallinity measurement technique developed by Yamamoto [42], with which crystallinity can be determined per layer, showed that in liquid paraffin, the layers closest to the graphene are already highly crystalline, while the rest of the paraffin is still in molten form. After cooling, the wax was organised in highly crystalline layers, with the wax molecules all aligned parallel to the graphene sheet.

Thermal conductivity was measured using two techniques. The equilibrium MD technique using Green–Kubo relations provided the qualitative confirmation that TC in the direction perpendicular to the graphene sheet was strongly reduced in the layered paraffin wax. Using the RNEMD technique developed by Müller-Plathe [30], TC was measured per region in the xy-planes. The aligned layers close to the graphene in the otherwise molten paraffin showed an increased TC, while, in the remaining (liquid) paraffin, the difference in the x- and y-components of TC was very small, as expected in an isotropic melt. The strong segregation of chain lengths in the composite with bidisperse wax resulted in a clear difference in TC between the regions containing mainly long and short chains, respectively. In polydisperse composite samples, the segregation is lower and correspondingly the differences in TC are smaller.

The layering in the wax implies an additional bottleneck for heat transport from graphene fillers into the paraffin and vice versa, in addition the limitation of interfacial thermal resistance between the two materials. The findings from this study provide a new direction for the optimisation of the TC of paraffin–graphene composites, suggesting to look into the functionalisation of the graphene surface.

Additionally, measurement techniques for TC in the direction perpendicular to the planes of strongly layered materials could be further looked into.

## Figures and Tables

**Table 1:** Thermal conductivity of all samples, per region. Measurements were done using the RNEMD method, in x- and y-direction. Averages of these two are shown, with the standard deviation indicating the anisotropy. All composites show a higher TC in the layers closest to the graphene. In molten state, this is due to alignment of these layers. In solid state, further increase in TC in the layers close to the graphene is caused mainly by molecular weight segregation, with the better-conducting longer chains being concentrated close to the graphene.

Wax Type	Phase	Distance from Graphene	TC [W/(m K)]
eicosane	liquid	first two layers (2 above, 2 below)	0.21 ± 0.06
		remaining paraffin	0.16 ± 0.01
	solid	first two layers (2 above, 2 below)	0.35 ± 0.07
		remaining layers	0.23 ± 0.04
bidisperse	liquid	first two layers (2 above, 2 below)	0.25 ± 0.06
		remaining paraffin	0.18 ± 0.01
	semi-solid	first four layers (4 above, 4 below)	0.30 ± 0.02
		remaining paraffin	0.17 ± 0.01
	solid	first four layers (4 above, 4 below)	0.28 ± 0.02
		remaining layers	0.13 ± 0.02
polydisperse	liquid	first two layers (2 above, 2 below)	0.19 ± 0.03
		remaining paraffin	0.17 ± 0.01
	solid	first two layers (2 above, 2 below)	0.21 ±0.04
		remaining layers	0.18 ± 0.03

## Data Availability

Raw MD data is available from M.B.

## Supplementary Materials

The supporting information can be downloaded at: https://www.mdpi.com/article/10.3390/polym15092175/s1.
