# Supplementary material for: Molecular Weight Segregation and Thermal Conductivity of Polydisperse Wax–Graphene Nanocomposites
Source: Polymers (Basel). 2023 May 3;15(9):2175. doi: 10.3390/polym15092175 (PMC10180893; doi:10.3390/polym15092175)
Supplement: Supplementary file 1 [file polymers-15-02175-s001.zip › polymers-2282120-supplementary.pdf]

Molecular Weight Segregation and Thermal Conductivity of Polydisperse Wax-Graphene Nanocomposites

Appendix A SUPPLEMENTAL INFORMATION

In the present study, graphene flakes are represented by infinite sheets crossing the periodic boundaries in x- and y-direction. Graphene nano-platelets (GNPs) used in practice are typically multiple micrometers in length and width (L. Wijkhuijs, personal communication, 23-03-2022), far larger than the dimensions of our simulation boxes of at most 10 nanometers. Having infinite graphene sheets means that the x- and y-dimensions of the simulation boxes should be held constant. The decrease of volume of the paraffin during cooling could therefore only be established by shrinkage of the simulation box in z-direction.

In the results discussed in the main part of this publication, it was observed that during crystallisation of the paraffin some empty space was formed in the simulation box due to this restriction of volume change. In Figure S1 an extreme example of such a void is shown. In these two layers, as shown from above, a clear void has formed in the crystallised monodisperse wax.

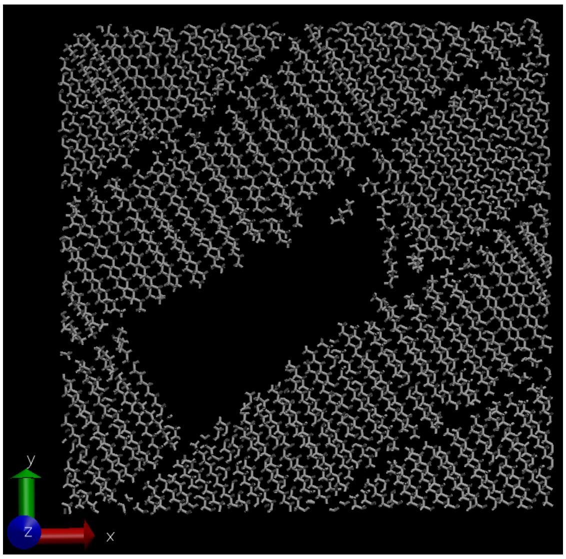

Figure S1: *Extreme example of a void in crystallised paraffin wax. Only two layers are depicted from above, showing clearly the void in the centre. This partial visualisation was taken from the composite with monodisperse eicosane.*

When the graphene sheet is made finite, the simulation box can be allowed to shrink in all dimensions to prevent formation of holes. As shown in Figures S2a and S2b, oriented layers

of paraffin wax are present close to the graphene flake in the crystallised wax, while further away the orientation of the paraffin is not parallel to the graphene. However, these graphene flakes have lengths roughly three orders of magnitude smaller than GNPs used in practice. This confirms the validity of representing of GNPs by infinite sheets.

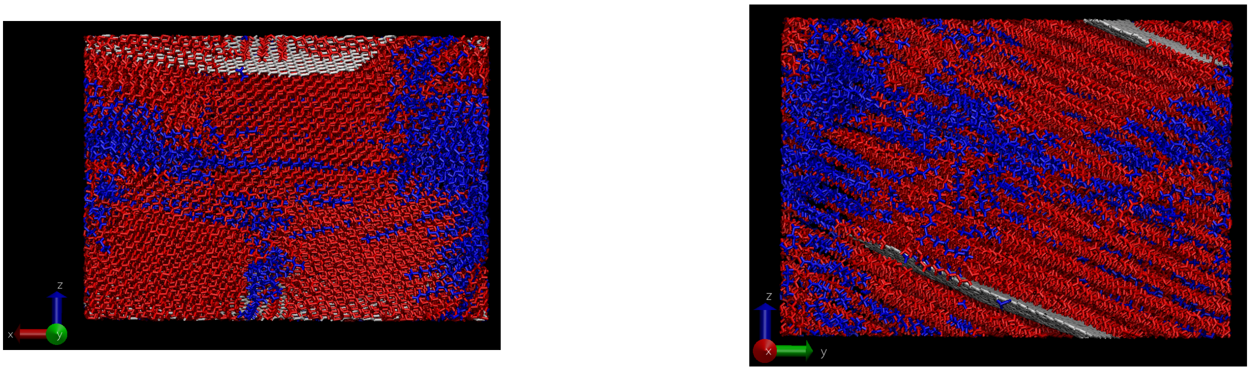

(a) (b)

Figure S2: (a) *View in y-direction on bidisperse paraffin containing a freely moving finite graphene flake, which is shown in white.* (b) *View in x-direction on te same simulation result. The decane chains, shown in blue, are not fully aligned with the graphene flake as was the case with the infinite graphene sheet discussed in the main results.*

Finally, a simulation with double the amount of paraffin compared to the simulations discussed in the main text was run. By combining the infinite graphene sheet with 1800 paraffin molecules, a twice as thick block of layered paraffin formed during cooling. As shown in Figure S3, the wax crystallises into aligned layers parallel to the graphene. Layers closest to the graphene are comprised mainly of the longer triacontane chains while the shorter decane molecules are concentrated in a region further away from the graphene. These observations were the same for the bidisperse composite with 900 paraffin molecules, from which we can conclude that 900 molecules suffice for this research.

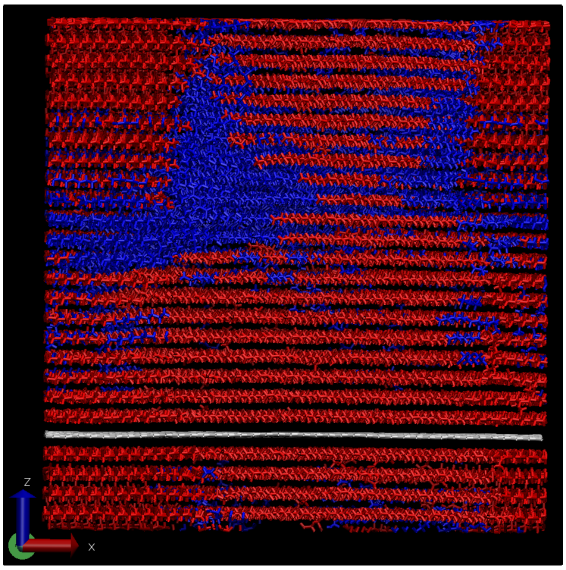

Figure S3: *Composite with bidisperse wax, containing 1800 paraffin molecules. Although the amount of wax is doubled compared to the 900 molecules in the main results, the alignment and segregation are still the same.*
